# Evaluating an obstetrics and gynecology teaching program for medical students incorporating simulation-based education underpinned by cognitive load theory

**DOI:** 10.3389/fmed.2024.1304417

**Published:** 2024-03-25

**Authors:** William Atiomo, Farah Ennab, Adrian Stanley, Mutairu Ezimokhai

**Affiliations:** College of Medicine, Dubai Healthcare City, Mohammed Bin Rashid University (MBRU) of Medicine and Health Sciences, Dubai, United Arab Emirates

**Keywords:** obstetrics, gynecology, teaching, medical students, simulation, cognitive load theory

## Abstract

Although there have been previous publications on curriculum innovations in teaching O&G to medical students, especially utilizing simulation-based education, there have been none, as far as we know, incorporating and evaluating the outcomes using cognitive load theory. The aim of this article was to describe the introduction, implementation, and evaluation of an innovative teaching program in O&G, incorporating simulation-based education, underpinned by cognitive load theory. Cognitive load is defined as the amount of information a working memory can hold at any one time and incorporates three types of cognitive load—intrinsic, extraneous, and germane. To optimize learning, educators are encouraged to manage intrinsic cognitive load, minimize extraneous cognitive load, and promote germane cognitive load. In these sessions, students were encouraged to prepare in advance of each session with recommended reading materials; to limit intrinsic cognitive load and promote germane cognitive load, faculty were advised ahead of each session to manage intrinsic cognitive load, an open-book MCQ practice session aimed to reduce anxiety, promote psychological safety, and minimize extraneous cognitive load. For the simulation sessions, the faculty initially demonstrated the role-play situation or clinical skill first, to manage intrinsic cognitive load and reduce extraneous cognitive load. The results of the evaluation showed that the students perceived that they invested relatively low mental effort in understanding the topics, theories, concepts, and definitions discussed during the sessions. There was a low extraneous cognitive load. Measures of germane cognitive load or self-perceived learning were high. The primary message is that we believe this teaching program is a model that other medical schools globally might want to consider adopting, to evaluate and justify innovations in the teaching of O&G to medical students. The secondary message is that evaluation of innovations to teaching and facilitation of learning using cognitive load theory is one way to contribute to the high-quality training of competent future healthcare workers required to provide the highest standard of care to women who are crucial to the overall health and wellbeing of a nation.

## 1 Introduction

The reproductive health of women determines a nation’s health and the health of its future generations. The Global Strategy for Women, Children, and Adolescents’ Health, agreed in 2016, emphasizes that all women have the right to the highest attainable standard of health and wellbeing, including the physical, mental, and social aspects of health (1). Nonetheless, in many parts of the world, women continue to experience a range of poor health outcomes including maternal death during pregnancy and childbirth, with 800 women dying each day globally in 2020 (2). High-quality training and continuing professional development of competent future healthcare workers are therefore crucial to the overall health and wellbeing of a nation, including its economic prosperity. Medical schools play a pivotal role in providing this required high-quality training to their medical students, to improve women’s health, particularly during the Obstetrics and Gynaecology (O&G) clerkship.

Unfortunately, several challenges in teaching O&G to medical students continue to limit the ability of medical schools to provide this required high-quality training in many parts of the world. These include a decrease in delivery suite experience (3), a decline in the number of opportunities for students to learn how to perform a pelvic examination (4) and male students are also more likely to experience gender bias from patients during their O&G clerkships, therefore limiting their learning opportunities (5). Variable clinical exposure in O&G depending on the available learning opportunities at differing hospital sites can result in a variation in student clinical skill acquisition. Therefore, there is a need for medical schools to develop innovative teaching programs in O&G, to address some of these challenges, and specifically to ensure that they can continue providing the required high-quality training to their students. Some potential solutions to the challenges of teaching O&G include simulation-based education (including surgical skills training), simulated patients, and inter-professional education (6). However, it is important that curriculum innovations are based on educational principles that have been proven to promote, rather than inhibit learning.

One such educational theory is the cognitive load theory (7). Human memory, which is key to learning, consists of two key aspects: short-term (or working) memory and long-term memory. Short-term memory is finite in capacity and duration, and only able to hold up to four to seven chunks of information at any one time (8). On the other hand, long-term memory is believed to be infinite in capacity (9). During the learning process, new information is first transferred into short-term memory from where it is processed and transferred into long-term memory for future retrieval. Cognitive load is defined as the amount of information working memory can hold at any one time. Cognitive load theory incorporates three types of cognitive load—intrinsic, extraneous, and germane. Intrinsic cognitive load is the mental effort required to process new information that is directly relevant to learning. Extraneous cognitive load includes distractors that take up space in short-term memory (noise, irrelevant material on PowerPoint slides, and negative emotions in the learner such as anxiety). Germane cognitive load is the mental effort put into the acquisition/development of schemas of information to be held in long-term memory. To optimize learning, educators are encouraged to manage intrinsic cognitive load, minimize extraneous cognitive load, and promote germane cognitive load (10). High scores on the germane cognitive load (self-perceived learning) scale have been reported to be predictive of higher academic performance (11). A recent publication (2023) highlights the growing recognition of cognitive load theory as an effective theory of instruction. This is underpinned by 40 years of research and is designed to advance what is known about how students learn and how instructional methods should be designed to promote learning effectively (12).

One example of how cognitive load theory has been used to improve educational outcomes is a study (13) which found that medical students randomly assigned to practice intravenous venous line insertion using progressive training from low- to mid- to high-fidelity simulators had a higher rating on global clinical performance, communication, and technical skills than those students who trained with either a low-fidelity or high-fidelity simulator alone. It is thought (14) that the complex cognitive processes involved in consolidating, retrieving, and transferring knowledge might not have been possible if the initial working memory processing was hindered by cognitive overload. Progressive learning was thought to result in better learning outcomes because by gradual knowledge building in a low- to high-complexity sequence, students were able to increase their knowledge stored in long-term memory and were ultimately able to tackle the highest complexity situations and gain the necessary exposure to the highest complexity. In another example (15), emergency medicine resident training in the classification of orthopedic fractures was investigated by randomizing the learners into two groups. One group involved active learning with the classification chart provided after each diagnostic answer submission. However, the other group were guided by forming a diagnosis by providing the classification chart with each diagnostic question. The latter optimized germane cognitive load, and this group had higher test scores and lower perceived overall cognitive load scores. With regard to simulation-based education, four other studies also demonstrate positive benefits from applying the principles of cognitive load theory. In the first study (16), researchers investigated the effect of implementing cognitive load theory-based design principles in virtual reality simulation training of surgical skills. They found that novice medical students who received cognitive load theory-based instructions had significantly increased cognitive load during post-training procedures compared to those who received standard instructions. This increased cognitive load was reflected in their performance, as the intervention group had a significantly lower final-product score than the control group. The second study (17) focused on ultrasound-guided internal jugular catheterization training. Using cognitive load theory principles, the researchers developed a curriculum incorporating progressive part practice in a simulation laboratory. They compared the technical proficiency of residents trained with this curriculum to those trained with a single simulation session. After three sessions, the experimental group showed significantly better hand motion and completion time scores compared to the control group. Even when assessed for retention at a later date, the experimental group still performed significantly better than the control group. In the third study (18), researchers used cognitive load theory principles to design a low-fidelity simulation (LFS) for the assessment and management of deteriorating patients. They measured the self-rated ability of undergraduate nurses in pre- and post-tests and found that their ability significantly increased after participating in the LFS. The fourth study (19) focused on using preparatory e-learning modules to improve performance in simulation-based education. The researchers developed online modules based on cognitive load theory and simulation-based education principles and assessed their impact on cognitive load and performance. Participants who received the online modules had higher intrinsic and germane cognitive load, and lower extraneous cognitive load during the course component compared to those who did not (control). During the simulation-based objective structured clinical examination, the online modules group performed significantly better than the control group. Overall, these studies highlight the importance of considering cognitive load theory principles in the design of simulation training. In theory, simulations, such as illustrative diagrams and video clips, are assumed to promote germane cognitive load though they may increase intrinsic cognitive load.

Although there have been previous publications on curriculum innovations in teaching O&G to medical students (20–23), especially utilizing simulation-based education, there have been none, as far as we know, incorporating and evaluating the cognitive load in O&G. The aim of this article was to describe the introduction, implementation, of an innovative teaching program in O&G that incorporates simulation-based education, aimed at addressing some of the current challenges in teaching O&G to medical students and evaluation of its cognitive loads and outcomes. We believe it is a framework that other medical schools might want to consider and to provide the high-quality training required to improve women’s health outcomes globally.

## 2 Methods

### 2.1 Learning environment and needs assessment

The Bachelor of Medicine Bachelor of Surgery (MBBS) program at Mohammed Bin Rashid University, Dubai, UAE (MBRU), is a 6-year undergraduate medical program divided into three phases. Phases 1 (Year 1) and 2 (Years 2 & 3) consist of basic sciences organ system courses to prepare the students for clinical clerkships in Phase 3 (Years 4–6) of the program. All students undertake a Year 3 three-credit Human Reproduction course, while the 8-week O&G clerkship takes place in Year 5.

The aim of the 8-week O&G clerkship is to familiarize students with the signs and symptoms of normal and abnormal reproductive function and to teach the basic examinations in O&G. This is achieved using a blended learning approach of face-to-face teaching on placements at both government and private hospitals, simulation sessions on the MBRU campus, and online resources, which include a study guide, range of videos, and revision material. The course aims to emphasize and reinforce skills for taking an appropriate history, performing a physical and pelvic examination, formulating a differential diagnosis as well as a treatment plan, and effectively managing patients. Students also undertake a 4-week O&G placement in the final (6th) year of the program. This takes the form of an apprenticeship which provides the students an opportunity to consolidate their knowledge, skills, and professional competencies in O&G, before graduating as doctors.

The first cohort of students were admitted in 2016. In early 2022, a review of the O&G clerkship was undertaken after the first cohort of students had completed their Year 5 clerkship. This involved several analyses. Mostly, a quantitative analysis was undertaken of formal student feedback scores for the first cohort. In addition, a qualitative (thematic) analysis was undertaken of free-text comments obtained from the students who were currently in Year 5 and had completed their O&G placements at the time of the review and a qualitative (thematic) analysis of free-text comments from a Year 6 student, reflecting on their experience of the O&G clerkship, which they completed in the 2020/2021 academic year. The quantitative analysis revealed that the key areas of strength were that ‘the clerkship objectives were clearly communicated at the beginning of the placement’ and that ‘teaching materials were provided in advance (when appropriate)’. However, the areas identified for improvement were that ‘formal teaching could be more relevant to the course objectives, feedback provided on students’ clinical performance could be timely and informative’ and ‘ensuring that the teaching in Phases 1 and 2 of the MBBS programs better prepared the students for the clinical clerkship’.

From qualitative analysis, the top positive theme was ‘good teaching and support of learning from the adjunct faculty’ and the top recommendation for improvement was the need for ‘centralized’ MBRU-based teaching (in contrast to teaching at individual hospital placement sites to fragmented clerkship sub-sets). Informal feedback from students and faculty identified a variation in opportunities for medical students to learn clinical skills relevant to O&G, such as pelvic and obstetric examination and delivery of a baby, depending on the location of the hospital placement and the students’ gender. A curriculum mapping exercise was also undertaken to identify knowledge and skills gaps in the Year 5 O&G clerkship: Core topics listed in the MBRU Year O&G 5 study guide (2021–2022) and faculty-recorded lectures on the MBRU learning management system were mapped to the Royal College of Obstetrics and Gynecology (RCOG) undergraduate curriculum available at the time of the exercise (24).

### 2.2 Pedagogical framework and format

The outcome of the evaluation exercise was communicated to the Dean of the medical school and other senior faculty with a proposal to address the issues identified. The proposal included the introduction of four 2- to 3-h MBRU-based centralized (for all students in the clerkship group) teaching sessions on weeks 1, 3, 5, and 7 of each 8-week clerkship rotation. The sessions were held on a Monday morning (aligning to the over-arching Year 5 curriculum delivery) and included an induction/introduction session on week 1 and sessions on labor and delivery (week 3), obstetric emergencies (week 5), and gynecology emergencies (week 7). The chosen topics addressed some of the issues highlighted in the student’s feedback and were core topics important to the curriculum of all O&G clerkships globally (25). Two revision sessions before each biannual (December and May) examination (1 for each half of the cohort) were also introduced. Table 1 shows a summary of a typical day of instruction for students in the new curriculum as compared to the old.

**Table 1:** Summary of a typical day of instruction for students in the new curriculum as compared to the old.

	Old curriculum				New curriculum
	Students in hospital site 1	Students in hospital site 2	Students in hospital site 3	Students in hospital site 4	All students
TIME/DAY	Monday	Monday	Monday	Monday	Monday
0:730–08:30	SELF STUDY	Ward Rounds/Handover	Handover with Hospitalist	Handover in Ward	*Scheduled Discipline Teaching at MBRU
08:30–12:00	Community Obstetrics and Gynecology Clinic	Join Registrar (resident) in ward round	Ward work tutorials	Ward (Student 2 and student 5), Antenatal clinic (Student 3), Operating theater (Student 1 and Student 4)	
13:00–16:30		Tutorial in hospital from 12:00–13:00 then MBRU longitudinal theme teaching afternoon from 14:00 to 17:00	MBRU longitudinal theme teaching afternoon from 14:00 to 17:00	MBRU longitudinal theme teaching afternoon from 14:00 to 17:00	MBRU longitudinal theme teaching afternoon from 14:00 to 17:00

^*^All students. From 09:00 to 12:00 h. MBRU, Mohammed Bin Rashid University.

**Figure 1:**
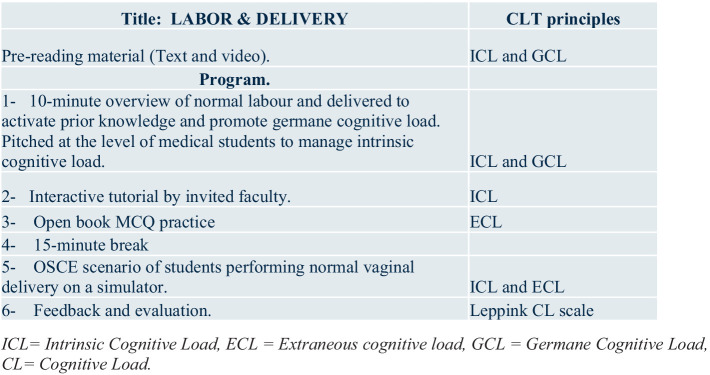
Description of the program and the application of the principles of cognitive load theory. ICL, Intrinsic cognitive load, ECL, extraneous cognitive load, GCL, germane cognitive load, CL, cognitive load.

Figure 1 illustrates a typical session and how the principles of cognitive load theory were applied. Students were encouraged to prepare in advance of each session where relevant, with recommended reading material communicated ahead of the sessions: This was designed to limit intrinsic cognitive load on the day of the session and promote germane cognitive load (8). Faculty were also advised ahead of each session to manage intrinsic cognitive load. The usual structure of the sessions included starting with a brief introduction and overview of the topic, followed by a 25-min interactive ‘flipped classroom’ discussion with the faculty. This was followed by a 20-min open-book MCQ practice session: These formative questions were relevant to the topic and written in a similar format to those used in their end-of-block examinations. The purpose of the open-book MCQ format, in contrast to closed-book MCQs, was to reduce anxiety, promote psychological safety, and minimize extraneous cognitive load (26). Students were permitted to discuss possible solutions in groups of two or three. This was followed by a 15-min break, after which the students engaged in a 45-min simulation scenario relevant to the topic of the day. The induction day included an introduction to history taking, pelvic examination, obstetric examination, and revision of key clinical O&G conditions which the students had been taught in their Year 3 Human Reproduction course. The scenarios for the induction session in week 1 included practice on task trainers on pelvic and obstetric examinations. During the labor and delivery session on week 3, each student performed a normal vaginal delivery on a task trainer. The obstetric emergency session on week 5 involved an OSCE scenario on the management of a patient with post-partum hemorrhage and the gynecology emergency session on week 7 involved an OSCE scenario of a simulated patient with an ectopic pregnancy. These scenarios start with the facilitating faculty, initially demonstrating the role-play situation or clinical skill being taught first, before asking the students to participate. This aimed to manage intrinsic cognitive load and reduce extraneous cognitive load (8), (27). The sessions concluded with a 10-min feedback and evaluation session during which students completed an online structured questionnaire and provided verbal instant feedback on their perceptions of how the sessions went. The revision sessions held before each biannual (December and May) examination, which involved students rotating through three mock OSCE scenarios on pelvic examination, obstetric examination, and gynecology history taking, on task trainers and a simulated patient, conducted in a similar style to the actual examination. Feedback was provided to students on their performance and general expectations during the final OSCE examinations.

The practicalities of implementing the sessions included communicating the plans to key faculty and liaising with key staff at the simulation center at MBRU to support delivering the sessions and timetabling. A meeting was held with the faculty facilitating each session, or an email was sent, the week before the session, to discuss the plan, during which cognitive load theory was discussed. Students did not have a formal teaching session on cognitive load theory as part of their education. However, on several occasions across the academic year, the principles of the theory were informally discussed with the students as part of the advice given to support their study skills. MBRU also holds an annual medical education meeting for faculty participating in the medical student teaching program during which the principles of cognitive load theory are discussed. The sessions were delivered during the 2022–2023 academic year with a slight modification following feedback from the first cohort of students for a need to consider introducing a 10-min brief overview of the topic by an in-house faculty member of the day in a didactic format at the start of each session.

### 2.3 Evaluation

The sessions were evaluated by analyzing the results of data obtained after students had anonymously completed an online structured questionnaire (Supplementary Figure S1 and Table 2). The questionnaire had a quantitative and qualitative domain. The quantitative domain was based on an expanded cognitive load scale (28) initially developed by Leppink et al. (29) and is one of the most validated and widely used self-report measures of intrinsic load (IL), extraneous load (EL), and germane load (GL). The introductory paragraph to the questionnaire informed students that the inventory measured their cognitive load during the teaching or revision session. They were advised to read each of the questions carefully and mark their responses to each question, on a rating scale from 0 (not all the case) to 10 (completely the case). The three components measured included the intrinsic cognitive load (ICL) of the sessions, the extraneous cognitive load (ECL), and the germane cognitive load or self-perceived learning (SPL) (11, 30). The qualitative domain asked, ‘Please feel free to include any free text comments below’.

**Table 2:** Student feedback questionnaire used.

Student feedback questionnaire used		
1	The topics covered in the session were very complex.	IL
2	The session covered theories that I perceived as very complex	
3	The session covered concepts and definitions that I perceived as very complex.	IL
4	The instructions and/or explanations during the session were very unclear.	EL Ins
5	The instructions and/or explanations during the session were in terms of learning very ineffective.	EL Ins
6	The instructions and/or explanations during the session were full of unclear language	EL Ins
7	Low quality audio made the instructions hard to follow.	EL Ins
8	Noises in the environment made it difficult to focus on the learning content.	EL Noi
9	Distractions in the environment made learning ineffective.	EL Noi
10	Unrelated events occurring in the environment made it difficult to focus.	EL Noi
11	My activities on my phone/computer made it difficult to focus on the learning content.	EL Dev
12	Messages and notifications from my phone/computer made learning unclear.	EL Dev
13	Others’ phone/computer use distracted me, making it hard to learn.	EL Dev
14	Technical issues made learning ineffective.	EL Dev
15	Problems with technology made it difficult to focus.	EL Dev
16	The session really enhanced my understanding of the topic(s) covered.	GL/SPL
17	The session really enhanced my knowledge and understanding of obstetrics and gynecology.	GL/SPL
18	The session really enhanced my understanding of the theories covered.	GL/SPL
19	The session really enhanced my understanding of concepts and definitions.	GL/SPL
20	Please feel free to include any free text comments below.	

IL, Intrinsic load; EL Ins, extraneous load instructions; EL Noi, extraneous load noises; EL Dev, extraneous load devices; GL, germane load; SPL, self-perceived learning.

The data were analyzed using Microsoft Excel. The means, standard deviations, median, and interquartile ranges were calculated for all the scores obtained in response to the questions relevant to the cognitive load domain. Specifically, all the scores in response to questions 1 to 3 in Table 2 (‘The topics covered in the session were very complex’, ‘The session covered theories that I perceived as very complex’, and ‘The session covered concepts and definitions that I perceived as very complex’) were used to calculate the mean and median intrinsic cognitive load (IL) imposed by the teaching sessions. The scores in response to questions 4–7 in Table 2 (‘The instructions and/or explanations during the session were very unclear’, ‘The instructions and/or explanations during the session were in terms of learning very ineffective’, ‘The instructions and/or explanations during the session were full of unclear language’, and ‘Low quality audio made the instructions hard to follow’) were used to calculate the mean and median extraneous cognitive load stemming from instructions (EL Ins). The scores in response to questions 8–10 in Table 2 (‘Noises in the environment made it difficult to focus on the learning content’, ‘Distractions in the environment made learning ineffective’, and ‘Unrelated events occurring in the environment made it difficult to focus’) were used to calculate the mean and median extraneous cognitive load stemming from noises (EL Noi). The scores in response to questions 11–15 in Table 2 (‘My activities on my phone/computer made it difficult to focus on the learning content’, ‘Messages and notifications from my phone/computer made learning unclear’, ‘Others’ phone/computer use distracted me, making it hard to learn’, ‘Technical issues made learning ineffective’, and ‘Problems with technology made it difficult to focus’) were used to calculate the mean and median extraneous cognitive load stemming from devices (EL Dev). Finally, the scores in response to questions 16–19 in Table 2 (‘The session really enhanced my understanding of the topic(s) covered’, ‘The session really enhanced my knowledge and understanding of obstetrics and gynecology’, ‘The session really enhanced my understanding of the theories covered’, and ‘The session really enhanced my understanding of concepts and definitions’) were used to calculate the mean and median germane cognitive load or self-perceived learning (GCL/SPL). The student’s responses to the qualitative domain asking, ‘Please feel free to include any free text comments below’, were analyzed with a natural language processing program on 3 July 2023 (31) with the prompt being ‘Provide a thematic analysis of the text in the following table’, with the responses of free-text students copied and pasted from the table of results into the prompt box, after the prompt. The natural language processing program used was ChatGPT. Developed by OpenAI, ChatGPT is a language model that utilizes natural language processing within the field of artificial intelligence. Its purpose is to provide text-based responses to queries in a manner that simulates human conversation. ChatGPT is constructed using the Transformer deep learning architecture, allowing it to recognize language patterns and generate coherent and realistic text. Through training on extensive text data, it has garnered the ability to generate responses across various subjects, ranging from basic inquiries to intricate conversational topics.

### 2.4 Institutional review board considerations

Formal institutional review board approval was not sought for the study because it was a desk-based retrospective review of student feedback data that did not involve any direct patient contact. The Medical Research Council (MRC) Regulatory Support Center/UK NHS Health Research Authority (HRA) online decision support tool^1^ does not classify the study as research. Nevertheless, the study was submitted to the chair of the MBRU institutional review board (IRB) committee for review, who provided written confirmation that the study was exempt and did not require their approval.

## 3 Results

The new centralized MBRU-based O&G teaching sessions were delivered from 23 August 2022 to 24 April 2023. There were 18 sessions in total, with four sessions (induction to O&G, labor and delivery, obstetric emergencies, and gynecology emergencies) delivered on the Monday morning of weeks 1, 3, 5, and 7 for each of the four, 8-week O&G clerkships over the academic year (16 in total) and two revision sessions before each biannual (December and May) examination (one session for each half of the cohort). In total, 48 students were in this Year 5 cohort; thus, 12 students rotated through each 8-week O&G clerkship. The maximum possible number of responses to the online structured questionnaire that students completed at the end of each session was 240 (16 sessions × 12 students and 2 sessions × 24 students): 206 responses were submitted and thus a response rate of 86%.

### 3.1 Students self-reported scores of cognitive loads

Table 3 presents the results of the students’ self-reported scores of cognitive load scales associated with the new O&G teaching sessions at MBRU. The mean (standard deviation (SD)) and median (interquartile range (IQ)) intrinsic cognitive scores from all sessions were 3.9 (2.9) and 3 (1-6) respectively. The mean and median scores of measures of extraneous cognitive load ranged from 1 to 1.5. The mean (SD) and median (IQ range) scores for measures of germane cognitive load or self-perceived learning on the cognitive load scale used to evaluate all the sessions were 9.5 (1.3) and 10 (10-10).

**Table 3:** Students self-reported scores of the different components of the cognitive load scale, associated with the O&G teaching sessions at MBRU.

	All sessions	Clerkship introduction	Labor & delivery	Obstetric emergencies	Gynecology emergencies	Revision
Mean ICL (SD)	3.9 (2.9)	3.5 (2.6)	4.4 (2.8)	4.5 (3)	3.4 (2.9)	3.5 (2.9)
Median ICL (IQ range)	3 (1–6)	2 (1–5)	4 (2–7)	3 (2–7)	2 (1–6)	2 (1–5)
Mean ECL INS (SD)	1.2 (1)	1.4 (1.6)	1.5 (0.5)	1 (0.8)	1.3 (1.3)	1.1 (0.3)
Median ECL INS (IQ range)	1 (1–1)	1 (1–1)	1 (1–1)	1 (1–1)	1 (1–1)	1 (1–1)
Mean ECL NOI (SD)	1.2 (1)	1.2 (1.4)	1.1 (0.6)	1.1 (0.4)	1.4 (1.40)	1.1 (0.2)
Median ECL NOI (IQ range)	1 (1–1)	1 (1–1)	1 (1–1)	1 (1–1)	1 (1–1)	1 (1–1)
Mean ECL DEV (SD)	1.1 (0.9)	1.3 (1.4)	1.0 (0.3)	1.1 (0.4)	1.3 (1.4)	1.1 (0.2)
Median ECL DEV (IQ range)	1 (1–1)	1 (1–1)	1 (1–1)	1 (1–1)	1 (1–1)	1 (1–1)
Mean GCL/SPL (SD)	9.5 (1.3)	9.3 (1.7)	9.4 (1.4)	9.7 (0.7)	9.4 (1.4)	9.7 (0.6)
Median GCL/SPL (IQ range)	10 (10–10)	10 (9–10)	10 (9.8–10)	10 (10–10)	10 (9–10)	10 (10–10)

The sessions that imposed the highest intrinsic cognitive load were those on obstetric emergencies and labor and delivery with mean scores of 4.5 (3) and 4.4 (2.8), respectively, and median scores of 3 (2-7) and 4 (2-7), respectively. The session that imposed the lowest intrinsic cognitive load was on gynecological emergencies with mean and median scores of 3.4 (2.9) and 2 (1-6) respectively. The session on obstetric emergencies, which imposed the highest intrinsic cognitive load, also had the highest measures of germane cognitive load or self-perceived learning with mean (SD) and median (IQ range) scores of 9.7 (0.7) and 10 (10-10) respectively. However, the revision sessions which were associated with low mean and median scores of intrinsic cognitive load 3.5 (2.9) and 2 (1-5) were also associated with the highest measures of germane cognitive load or self-perceived learning with mean (SD) and median (IQ range) scores of 9.7 (0.6) and 10 (10-10), respectively.

### 3.2 Thematic analysis of student’s free-text comments

The results of the thematic analysis of the student’s free-text feedback are presented in Table 4. Overall, the thematic analysis found a positive response to the sessions, highlighting their efficacy in preparing participants for their rotations, reinforcing knowledge, and providing hands-on experience. Participants also provided valuable suggestions for improvement to enhance future sessions.

**Table 4:** Thematic analysis of student’s free-text comments:

1 Appreciation and gratitude: Many participants expressed their gratitude and appreciation for the sessions, citing how helpful and beneficial they were in preparing them for their rotations or exams. They thanked the organizers and instructors for their efforts.
2 Preparation and knowledge reinforcement: Participants mentioned how the sessions helped them prepare for their rotations, solidify their theoretical knowledge, and apply their learning in practical scenarios. They highlighted the importance of gaining confidence and being more comfortable in clinical settings.
3 Suggestions for improvement: Some participants offered suggestions for improvement, such as adding more time for practice, including visual aids or videos to enhance learning, and providing clearer guidelines or instructions.
4 Hands-on experience and interactive learning: Participants valued the hands-on experience and interactive nature of the sessions, which involved simulations and discussions. They appreciated the opportunity to practice different roles, receive feedback, and engage in case-based scenarios.
5 Constructive criticism: While most participants have positive feedback, a few provided constructive criticism regarding aspects such as visibility during demonstrations, the need for more formal teaching sessions, or the inclusion of specific visual aids to enhance the learning experience.
6 Positive impact on confidence and skills: Many participants expressed how the sessions enhanced their confidence, knowledge, and clinical skills. They mention feeling more prepared to assist during deliveries, handle emergencies, or approach certain conditions.
7 Organization and time management: Several participants appreciated the organization and structure of the sessions. They highlighted the importance of effective time management and proper planning, including the allocation of sufficient time for each activity.
8 Reinforcing theoretical concepts: Many participants emphasized the relevance of bridging theory and practice. They mention the benefits of discussing concepts, clarifying doubts, and reinforcing theoretical knowledge through practical application.

## 4 Discussion

The results of the evaluation of this new teaching program in O&G described in our study found that the students perceived that they invested relatively low mental effort in understanding the topics, theories, concepts, and definitions discussed during the sessions. There was low extraneous cognitive load because of the nature of the instructions or distractions from noise, or electronic devices (phone, computer, or technical issues) and measures of germane cognitive load or self-perceived learning were high. Students expressed appreciation for the organized and practical nature of the sessions, as well as the guidance provided by instructors. Areas for improvement were also identified, such as incorporating additional teaching materials and allowing for more time for practice. These changes have already been implemented for the subsequent academic year (2023–2024).

Although no previous study had measured cognitive load during simulation teaching of O&G, one study (30) measured cognitive load in 41 Year 5 (final year) students undergoing simulation teaching in a medical and surgical scenario. The median intrinsic cognitive load was slightly lower (score of 3) in our O &G scenario study compared with the scores (3.7 to 4.2) in the medical and surgical scenario study (23). The median self-perceived learning scores in our O&G scenario study were higher (score of 10) than the scores (6 to 6.8) in the study of medical and surgical scenarios (30), but the median extraneous cognitive load scores were similar in both studies (1 and 0.9). As high scores on the self-perceived learning scale have been reported to be predictive of high academic performance (11), these results could be interpreted as better achievement of learning outcomes in our study. On the other hand, the different results could be due to different designs of simulation teaching or the context for the studies. It is possible that the The low intrinsic cognitive load scores observed in our study might be attributed to the fact that these sessions occurred in parallel with clinical placements. Thus, students interact with real patients and problems, which may have prepared them for learning, as well as receiving pre-reading material ahead of the sessions. The location of the sessions outside hospital placements and within a dedicated simulation learning environment may have reduced extraneous cognitive load alongside the other measures we adopted during the sessions (e.g., open-book MCQs) to reduce this. Effective learning, as reflected in the high self-perceived learning/germane cognitive load scores, might also have taken place because of the mindset and the multiple methods of teaching, including simulation.

The sessions on obstetric emergencies and labor and delivery imposed the highest intrinsic cognitive load (a higher mental effort invested in learning) as the students may have found these tasks ‘complex’. These were OSCE-style scenarios simulating the clinical environment. The first was on a patient with post-partum hemorrhage and the second required the student to perform a vaginal delivery on a task trainer. These findings are consistent with previous research showing that simple tasks help students gain more self-confidence (32) but that complex simulated clinical environments impose greater intrinsic and extraneous cognitive load and stress on students. In contrast, the session that imposed the lowest intrinsic cognitive load was on a gynecological emergency, which involved a simulated OSCE scenario of a patient with an ectopic pregnancy. The latter was undertaken in week 7, and thus, students may have become more familiar with the style of teaching or the scenario was not perceived as complex as the earlier simulations.

Moreover, it was intriguing that there were high scores of self-perceived learning in the ‘complex’ sessions on obstetric emergencies and labor and delivery, which had imposed the highest intrinsic cognitive load. This does not quite fit what we would have expected; however, a recent randomized controlled trial in simulation-based teaching might provide some insight. This found that environmental complexity contributes to intrinsic cognitive load, but students seemed to strategically manage their own cognitive load and learn from these simulations (33). On the other hand, the associated drama, anxiety, and excitement during the simulation sessions on labor and delivery, could have made it a challenge to identify the specific learning outcomes. This infers that the relationship between intrinsic cognitive load, environmental complexity, and learning gained may not be straightforward. Thus, more research is required to clarify the link between task complexity, cognitive load, and learning in simulation-based teaching. It may be the case that increased intrinsic cognitive load is a ‘price to pay’ for acquiring germane cognitive load in simulation. However, from a practical perspective, good practice would be for task complexity to be adapted to the expertise level of the learners and increased progressively as they become more competent.

The expanded scale in our student evaluation used a multidimensional conceptualization of the extraneous load construct that was relevant to physical and online teaching environments. The expanded Leppink cognitive load scale includes items related to instructions/explanations with sub-dimensions, including extraneous load stemming from noises, and extraneous load stemming from both media and devices within the environment. The Leppink scale was used because of its wide use and its perceived ability to measure cognitive load in more realistic learning environments, consistent with the learning environment of our students. We were also mindful that teaching with clinical simulation can induce both emotional and cognitive overload (30) and were keen to objectively evaluate this.

The findings from the needs assessment prior to introducing the new program are consistent with previous research showing that core knowledge and competencies acquired during O&G clerkships vary widely depending on the medical school (25). The call for a need to ensure that teaching in the earlier phases of the MBBS program should better prepare them for the clinical clerkship is consistent with the finding that clinical reasoning requires knowledge in real or simulated clinical environments (34). This matter was addressed by introducing a formal induction session, which included an introduction to history taking and revision of core O&G clinical conditions, which the students had previously been taught during the Year 3 Human Reproduction course.

The challenge of gender bias against male students, which was one of the factors leading to the curricular reforms in this study, should hopefully be addressed by this new program. As these barriers might persist post-qualification, it is important that postgraduate educational programs (residency and continuing professional development) also explore innovative educational curricula to ensure high-quality training and professional development to address this challenge. It is interesting to note that in a systematic review of 15 studies (35), patients prioritized their physician’s care, technical skills, compassion, and experience over gender when choosing their obstetricians and gynecologists. Therefore, barriers to learning because of gender bias may be less of a challenge for practicing doctors compared with medical students.

The strengths of this innovative program curriculum in teaching O&G to medical students are in the incorporation of cognitive load theory in the design, implementation, and evaluation. The process was also consistent with the recommended steps of curriculum development (36). The format used in the sessions was rated highly by students in a previous publication (20). To adapt this format further, we were, however, mindful of underpinning our sessions with principles of cognitive load theory (37).

There were some limitations. This included the lack of a control group, lack of student assessment data to objectively measure learning (examination results), response bias, and potentially limited generalizability.

It was difficult to develop an appropriate and ethical control group because of the nature of the study. Moreover, the complexity of the intervention would have made it a challenge to develop a control group; for example, the type of cognitive load addressed shifted with different aspects of the new curriculum. There were multiple interventions. Students were encouraged to prepare in advance of each session with recommended reading materials; this was aimed to limit intrinsic cognitive load and promote germane cognitive load, mindful that the teaching would involve a brief introduction followed by a ‘flipped classroom’ discussion. In addition, faculty were advised ahead of each session to manage intrinsic cognitive load during the 25-min tutorial. The format of the 20-min open-book MCQ practice session aimed to reduce anxiety, promote psychological safety, and minimize extraneous cognitive load. For the simulation sessions, the faculty initially demonstrated the role-play situation or clinical skill first, which aimed to manage intrinsic cognitive load and reduce extraneous cognitive load. It would therefore have been challenging to determine which specific component of the intervention worked, even with a control group. The feasibility and resource implications of a systematic variation in the various components of the intervention (e.g., types of cognitive load) to determine which component worked, in a study with a control group or groups would also have been challenging, and particularly so with a small cohort of less than 50 students. However, as proof of concept in our setting, this study has provided preliminary data to inform the design of future studies.

With respect to assessments, objective measures of learning outcomes, such as summative examination results, could provide more robust evidence of the effectiveness of the program. However, the student feedback in the study was collected anonymously and it was not possible to link responses from individual students to their examination results. The O&G summative examination results from both the cohort of 48 students who underwent the novel teaching program and the cohort in the previous academic year (34 students) were analyzed: There was no significant difference in either OSCE or theory results. This is not surprising. There are challenges with assessing the effectiveness of any new teaching program because of confounding variables. Some of these include test anxiety, variation in marking standards, student motivation, student social interactions outside of the classroom, and the student’s independent study. Furthermore, the sessions only covered a part of the syllabus, whereas the O&G examination covered wider aspects. Longitudinal assessment data might have also provided insights into the sustainability of the effects of program on students’ learning outcomes and clinical performance. However, this was not possible as the data were obtained from the students anonymously.

As the study was conducted at a specific institution, this limits the generalizability of the proposed teaching program to other medical schools. Resource limitations may also impact the feasibility and scalability of this innovative teaching program to other medical schools, especially those in low-resource settings, as building simulation centers with high-fidelity manikins require modern and expensive equipment (38). On the other hand, as long as the theoretical principles of cognitive load theory underpin the program, creative solutions (39) such as simulated patients and part-task trainers could provide a starting point in low-resource settings.

Feedback mechanisms might be biased toward students who are more vocal or have stronger opinions. However, our survey response bias was unlikely given the high response rate (86%) to the student evaluation questionnaires.

The order in which the questions were asked in the survey tool used may also have influenced the results, as it has been shown that asking learners about their intrinsic cognitive load (ICL) first, makes them give higher ICL ratings compared to asking them about their extraneous cognitive load (ECL) first (40). Finally, as far as we know, there is not a defined acceptable cutoff value for the different types of cognitive load we measured using the cognitive load measurement scale we used in our study. However, on a rating scale of 1–10, the mean and median scores of 9.5 and 10, of germane cognitive load or self-perceived learning, demonstrated in our study were consistent with positive learning outcomes.

In conclusion, the introduction, implementation, and evaluation of this innovative way of teaching O&G to medical students, underpinned by cognitive load theory, demonstrated positive outcomes. It is a model that other medical schools globally might want to consider, raise standards in teaching O&G (and other subjects) to medical students, and address some of the current educational challenges facing the teaching of O&G. The promotion of learning using cognitive load theory in the students who attended these sessions, should hopefully, contribute to the high-quality training of sufficient future healthcare workers required to provide the highest standard of care to women who are crucial to the overall health and wellbeing of a nation.

## Data availability statement

The raw data supporting the conclusions of this article will be made available by the authors, without undue reservation.

## Author contributions

WA: Conceptualization, Data curation, Formal analysis, Methodology, Project administration, Supervision, Writing – original draft, Writing – review & editing. FE: Project administration, Supervision, Writing – review & editing. AS: Writing – review & editing, Formal analysis. ME: Project administration, Supervision, Writing – review & editing.

## Footnotes

^1^http://www.hra-decisiontools.org.uk/research/ accessed on 4 July 2023
