# Supplementary material for: Evaluating an obstetrics and gynecology teaching program for medical students incorporating simulation-based education underpinned by cognitive load theory
Source: Front Med (Lausanne). 2024 Mar 25;11:1304417. doi: 10.3389/fmed.2024.1304417 (PMC10999601; doi:10.3389/fmed.2024.1304417)
Supplement: Supplementary file 1 [file Data_Sheet_1.PDF]

Evaluation of Session

Dear student,

This inventory measures your cognitive load during the teaching or revision session. All of the following questions refer to the session that has just finished. Please take your time to read each of the questions carefully and please mark your response on the form based on the following rating scale:

Not all the case Completely the case

0 1 2 3 4 5 6 7 8 9 10

The contents of this form were developed and validated by Dr. Jimmie Leppink from Department of Educational and Research, Faculty of Health, Medicine and Life Sciences, Maastricht University, Netherland and Martin S. Andersen and Guido Makransky from the Department of Psychology, University of Copenhagen, Copenhagen, Denmark.

1. Title of the session

2. Date

3. The topics covered in the session were very complex.

1	2	3	4	5	6	7	8	9	10

4. The session covered theories that I perceived as very complex

1	2	3	4	5	6	7	8	9	10

5. The session covered concepts and definitions that I perceived as very complex.

1	2	3	4	5	6	7	8	9	10

6. The instructions and/or explanations during the session were very unclear.

1	2	3	4	5	6	7	8	9	10

7. The instructions and/or explanations during the session were in terms of learning very ineffective.

1	2	3	4	5	6	7	8	9	10

8. The instructions and/or explanations during the session were full of unclear language

1	2	3	4	5	6	7	8	9	10

9. Low quality audio made the instructions hard to follow.

1	2	3	4	5	6	7	8	9	10

10. Noises in the environment made it difficult to focus on the learning content.

1	2	3	4	5	6	7	8	9	10

11. Distractions in the environment made learning ineffective.

1	2	3	4	5	6	7	8	9	10

12. Unrelated events occurring in the environment made it difficult to focus.

1	2	3	4	5	6	7	8	9	10

13. My activities on my phone/computer made it difficult to focus on the learning content.

1	2	3	4	5	6	7	8	9	10

14. Messages and notifications from my phone/computer made learning unclear.

1	2	3	4	5	6	7	8	9	10

15. Others' phone/computer use distracted me, making it hard to learn.

1	2	3	4	5	6	7	8	9	10

16. Technical issues made learning ineffective.

1	2	3	4	5	6	7	8	9	10

17. Problems with technology made it difficult to focus.

1	2	3	4	5	6	7	8	9	10

18. The session really enhanced my understanding of the topic(s) covered.

1	2	3	4	5	6	7	8	9	10

19. The session really enhanced my knowledge and understanding of obstetrics and gynaecology.

1	2	3	4	5	6	7	8	9	10

20. The session really enhanced my understanding of the theories covered.

1	2	3	4	5	6	7	8	9	10

21. The session really enhanced my understanding of concepts and definitions.

1	2	3	4	5	6	7	8	9	10

22. Please feel free to include any free text comments below.

This content is neither created nor endorsed by Microsoft. The data you submit will be sent to the form owner.

Microsoft Forms
